# A high-performance electrochemical biosensor using an engineered urate oxidase†

**DOI:** 10.1039/d3cc01869e

**Published:** 2023-05-30

**Authors:** Zheng Wei, Tanja Knaus, Yuxin Liu, Ziran Zhai, Andrea F. G. Gargano, Gadi Rothenberg, Ning Yan, Francesco G. Mutti

**Affiliations:** a Van 't Hoff Institute for Molecular Sciences, University of Amsterdam Science Park 904 1098 XH Amsterdam The Netherlands f.mutti@uva.nl

## Abstract

We constructed a high-performance biosensor for detecting uric acid by immobilizing an engineered urate oxidase on gold nanoparticles deposited on a carbon-glass electrode. This biosensor showed a low limit-of-detection (9.16 nM), a high sensitivity (14 μA/μM), a wide range of linearity (50 nM–1 mM), and more than 28 days lifetime.

## Abstract 2

We constructed a high-performance biosensor for detecting uric acid by immobilizing an engineered urate oxidase on gold nanoparticles deposited on a carbon-glass electrode.

## Graphical abstract

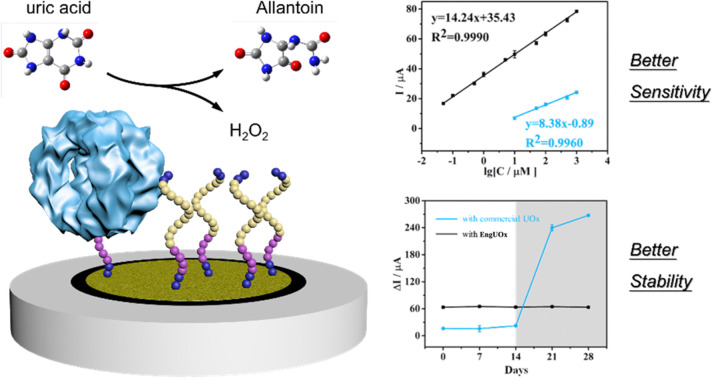

Biosensors based on oxidoreductase enzymes have been intensively studied during the last decade due to the requirement for more rapid and precise diagnosis protocols.^1–3^ However, naturally available (*i.e.*, wild-type) oxidoreductases are often unstable at high temperatures, leading to narrower operating conditions of the enzyme-based biosensing platforms and restricted applications.^4,5^ Therefore, several methods were proposed for increasing the thermal stability of enzymes within biosensors. The most frequently employed strategy in the field of biosensing consists in the encapsulation of the enzymes into porous network materials or the immobilization on nanofibers.^6,7^ The confinement of the enzymes can physically protect them from the outside hyperthermia and reduce their exposure to the potentially harsh environment. Furthermore, the structural stability of enzymes can be enhanced through coordination and intermolecular interactions inside the cage.^8^ However, the protection conferred by these materials has the disadvantage to decrease the actual catalytical activity of enzymes due to the reduced accessibility of analytes and the possible occupation of the active sites by the inner materials.^9^ Consequently, this methodology sacrifices catalytic performance to improve thermal stability, which is not ideal for the development of more efficient biosensing platforms. In view of this, a rational strategy would be expected that can endow enzymes with both high thermal stability and outstanding catalytical activity.

Structural-guided enzyme engineering with the aid of computational methods or the directed evolution of an enzyme can provide a solution to this dilemma by increasing the enzyme stability (*e.g.*, thermal, pH, additives) while retaining of even enhancing the catalytical efficiency.^10–14^ The specificity of the enzyme toward a substrate or a cofactor can also be optimized along with its regioselectivity, and stereoselectivity in some catalytic reactions.^15,16^ Thermal stabilization can also be enhanced by introducing salt bridges and/or disulfide bonds.^17^ Thus, protein engineering can improve the catalytic efficiency of enzymes for existing as well as new electrochemical biosensors.

Here, we constructed an electrochemical biosensor that harnessed the performance of a thermostable engineered enzyme, which was further modified in our group by genetically fusing a poly-histidine purification tag. As the essential biomarker for gout, uric acid (UA) was selected as the model analyte and we used urate oxidase (UOx) as the model enzyme in this study.^18,19^ UOx catalyzed the oxidation UA to yield H_2_O_2_^20^ as a secondary product, which then promoted the covalent immobilization of bovine serum albumin (BSA) onto electrodes through the formation of the disulfide bond between BSA and glutathione (GSH, see Scheme 1 and Scheme S1, ESI†).^21^

**Scheme 1:**
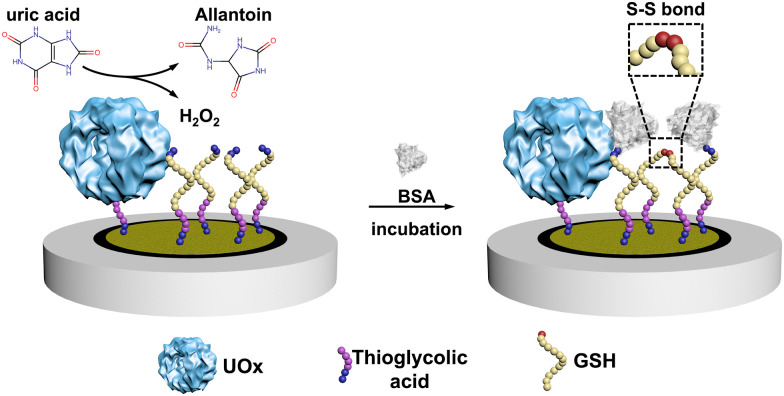
The principle of the electrochemical UA biosensor.

The engineered UOx from *Bacillus* sp. TB-90 (BTUO R298C, herein noted as engUOx) was recombinantly produced in *E. coli* and purified by Ni^2+^-affinity chromatography in our group (Fig. S1, ESI†).^4,22^ The reported substitution of the arginine 298 residue with a cysteine enables the spontaneous formation of an inter-subunit disulfide bond and significantly improved the thermo-stabilization of the enzyme (Fig. 1A). Additionally, this single point mutation did not result in any negative influence on the catalytical performance and eliminated the need for adding sulfate salt to stabilize the enzyme,^17^ thus improving the biosensor's reliability in our work. Therefore, the engUOx became an excellent candidate for constructing an advanced UA biosensor.

**Fig. 1:**
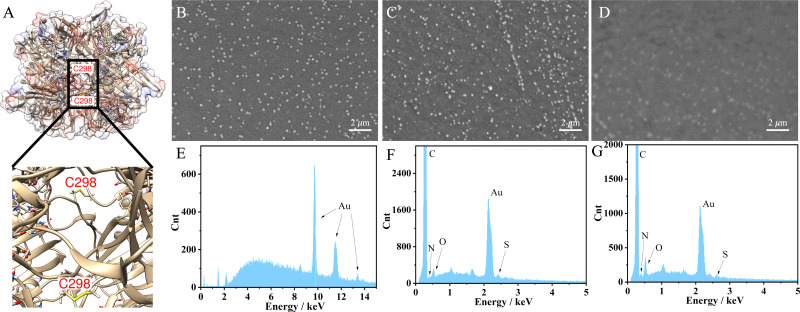
Structure of engUOx and characterizations of the biosensor. (A) 3D structure of engUOx highlighting the inter-subunit disulfide bonds. SEM images of electrochemically deposited AuNPs on GCE (B), incubated with engUOx (C), and incubated with UA and BSA (D). EDS images of electrochemically deposited AuNPs on GCE (E), incubated with engUOx (F), and incubated with UA and BSA (G).

After producing the enzyme, we built the electrochemical UA biosensor in a stepwise manner (Scheme 1, for more details see Scheme S1 in the ESI†). We deposited the gold nanoparticles (AuNPs) on the electrode by electroreduction as developed in previous work.^23^ Next, thioglycolic acid was added, forming Au–S bonds. The thioglycolic acid molecules, upon activation of the carboxyl groups, served as linkers to the engUOx by forming amide bonds. To further amplify the electrochemical signal, the electrode was decorated with sulfhydryl groups by attaching GSH, and BSA was selected as the impedance probe. When the UA is added, engUOx catalyzes UA oxidation and yields H_2_O_2_ as a by-product. This H_2_O_2_ reacts further, generating a disulfide bond between BSA and GSH, changing the conductivity and thereby the current of the electrochemical UA biosensor.

This stepwise process was characterized to ensure the successful implementation. Scanning transmission microscope (SEM) image clearly showed the deposited AuNPs on the surface of the electrode with a uniform distribution (Fig. 1B) while the image became less defined upon incubation with the engUOx due to the reduced conductivity of the electrode's surface (Fig. 1C). The energy dispersive X-ray (EDS) spectrum also evidenced the successful immobilization of engUOx, showing two new peaks of N and S atoms from the enzyme (Fig. 1F), which are not detected for the pure Au-deposited electrodes (Fig. 1E). Fourier transform infrared spectroscopy (FTIR) results also supported the successful construction of the biosensor (Fig. S2, ESI†). After attaching the BSA, the SEM image became blurry (Fig. 1D) and the ratio of the intensity of the N and S peaks *versus* Au peak in EDS spectrum increased (Fig. 1G). This shows that more protein was anchored on the electrode's surface. In the FTIR spectrum, we could observe the disulfide bond that confirmed the occurrence of the reaction between BSA and GSH in the presence of H_2_O_2_ (Fig. S2, ESI†). In a separated experiment, we also demonstrated that the BSA can be linked to GSH by the oxidative reaction in presence of H_2_O_2_. In fact, when BSA (66428 Da) and GSH were incubated in presence of H_2_O_2_, a molecular weight increase was observed by size-exclusion chromatography-mass spectrometry (SEC-MS: BSA + GSH 66734 Da; Fig. S3, ESI†).

The characterization of the stepwise procedure for the fabrication of the UA biosensor was also performed electrochemically by cyclic voltammetry (CV, Fig. 2A). Compared to bare electrodes, the electrodes deposited with AuNPs had higher current response because AuNPs are good conductive materials. After the covalent derivatization with the thioglycolic acid, the current value decreased due to the poor conductivity of the added organic molecule, thus demonstrating that the thioglycolic acid was successfully linked to AuNPs. Next, the similar behavior was observed after engUOx and GSH were immobilized on electrode, respectively. These observations confirmed that engUOx and GSH were successfully immobilized on the electrodes.

**Fig. 2:**
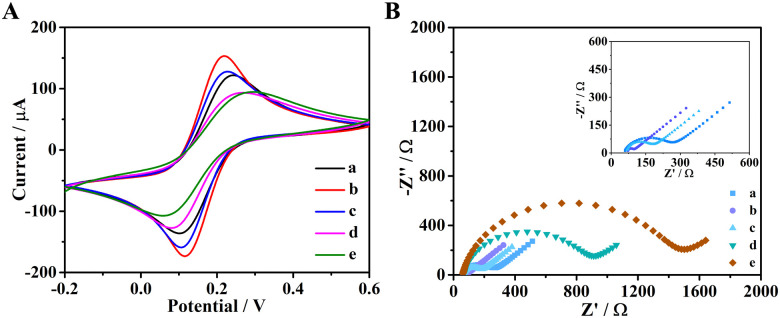
CV (A) and EIS (B) of different modified electrodes: bare electrode (curve a), AuNPs/GCE (curve b), thioglycolic acid/AuNPs/GCE (curve c), incubated with engUOx (curve d), blocked with GSH (curve e).

To further characterize the construction procedure, we conducted electrochemical impedance spectroscopy (EIS) to monitor the impedance of the biosensing interface (Fig. 2B). The non-Ohmic charge transfer impedance was monitored by quasi-semicircle diameter of the Nyquist plots. Compared with bare electrode (curve a), the electrodes decorated with AuNPs (curve b) had smaller diameter due to the good conductivity of gold material. After incubating with thioglycolic acid, the diameter of the semicircle increased a little (curve c), which indicated that thioglycolic acid was successfully linked on the electrodes. The impedance of the biosensing platform increased gradually upon engUOx (curve d) and GSH (curve e) immobilization. This electrochemical characterization also indicated that the UA biosensing platform was successfully constructed.

We then studied the influence of different parameters on the performance of the biosensor, comprising concentration of thioglycolic acid and glutathione, incubation time for them, and the catalytic reaction time (Fig. S4, ESI†). Judging by the square wave voltammetry (SWV) response, the obtained optimum conditions were used for the continuation of this work (see ESI,† Section S1.5).

We quantitatively evaluated the sensing performance with a specific focus on the limit of detection (LOD), linear detection range, selectivity, and reliability. The current in square wave voltametric test (SWV) decreased at increasing concentration of UA (Fig. S5, ESI†) and a linear relationship was observed between the current difference value (Δ*I*) and the logarithm of the UA concentrations in the range of 0.05–1000 μM (Fig. S6, ESI†). This linear correlation between the logarithm of UA concentration and the Δ*I* value is explained by the same type of relationship between the electrodynamical potential and the activity of the redox species in solution in the Nernst equation. Furthermore, this type of electrochemical data are fitted more appropriately after logarithmic transformation as recently reviewed.^24^ According to the literature data, the concentration of uric acid in the serum of healthy individuals ranges from *ca.* 210 μM to *ca.* 430 μM for adult men and from *ca.* 150 μM to *ca.* 360 μM for adult women. Uric acid concentration above these thresholds are critical for the diagnose of renal hyperuricemia and gout.^25^ In other physiological samples like sweat, the concentration of uric acid was determined to be as *ca.* 25 μM.^26^ Therefore, we can conclude that the biosensor developed in this work is suitable for determination of uric acid concentration in real samples. The LOD was calculated to be 9.16 nM, comparable with other UA biosensors described in previous reports. Linearity range and LOD values for the most common UA biosensors are reported in ESI,† Table S1 (and references herein). Notably, biosensors possessing a similar LOD value exhibit a narrower range of linearity, thus making them not broadly applicable for the determination of uric acid in real serum samples (ESI,† Table S1 entries 1 and 7). Moreover, owing to the characteristic specificity of the enzymes, our biosensor showed outstanding selectivity to UA in presence of different interferents (Fig. 3A and ESI,† Table S2). Since our UA biosensor operates with BSA as impedance probe, a sample containing only BSA produced the highest current of *ca.* 120 μA, which can be considered as the background. Samples containing BSA and each of the interferents at 1 mM concentration produced a negligible variation of the current, likely due to a small variation of the non-specific BSA absorption on the electrode's surface as known in the literature.^27–29^ In contrast, the UA sample measured at 10-fold lower concentration (100 μM) produced a great decrease of the current value (*ca.* 60 μA). Moreover, a sample obtained by mixing all the interferents (1 mM, each) with UA (100 μM) produced the same current as for a sample with UA alone (100 μM). These data demonstrates the high selectivity over competition for our UA biosensor, in agreement with the literature.^30^ Finally, the reliability of our biosensor was evidenced by measuring standard samples, since a regression coefficient of 0.9998 was calculated (Fig. 3B).

**Fig. 3:**
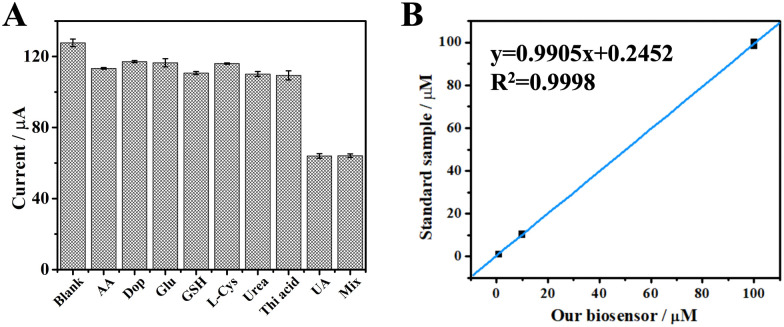
(A) Current responses measured to assess the specificity of the UA biosensor. The concentration of the interferent was set at 1 mM in each sample (AA: ascorbic acid; Dop: dopamine; Glu: glutamic acid; l-Cys: l-cysteine; Urea; Thi acid: thioglycolic acid). The concentration of uric acid sample (UA) was set at 100 μM. The mixture sample (Mix) consisted of all the interferents (1 mM, each) and UA (100 μM). Blank is a sample containing only BSA. (B) The concentration result obtained from our biosensor *vs* standard samples. The error bars are standard deviations for *n* = 3.

To prove the advantage of using engUOx within our biosensing platform, we constructed the same type of biosensor using the same procedure but with a commercial UOx (a wild-type enzyme from *Candida* sp.). Fig. 4 depicts the comparison of the biosensing performance between engUOx and the commercially available UOx. Upon incubation with UA solution, we measured a Δ*I* current value decreased of 15.44 μA for commercial UOx-based biosensor and of 63.80 μA for engUOx-based biosensor (Fig. 4A and B). These results implied that engUOx leads to a greater current change than commercial UOx in electrochemical biosensing process at the same conditions. We infer the higher sensing performance of engUOx compared with the commercial UOx to the higher stability of the former, so that a higher fraction of the enzyme can retain a high catalytic activity during the incubation. In fact, the engUOx used in this work originates from *Bacillus* sp. that is a thermostable soil bacterium.^17^ In contrast, the commercial UOx originates from *Candida* sp. that is a yeast.^31^ Enzymes from thermophilic bacteria are commonly more stable than from eukaryotes. Furthermore, engUOx was further stabilized by adding a sulfur-sulfur inter-subunit bridge.^17^ Finally, engUOx and the commercial UOx have a low sequence identity (24%) that supports the different catalytic behavior (for sequence alignment: ESI,† Section S1.10).

**Fig. 4:**
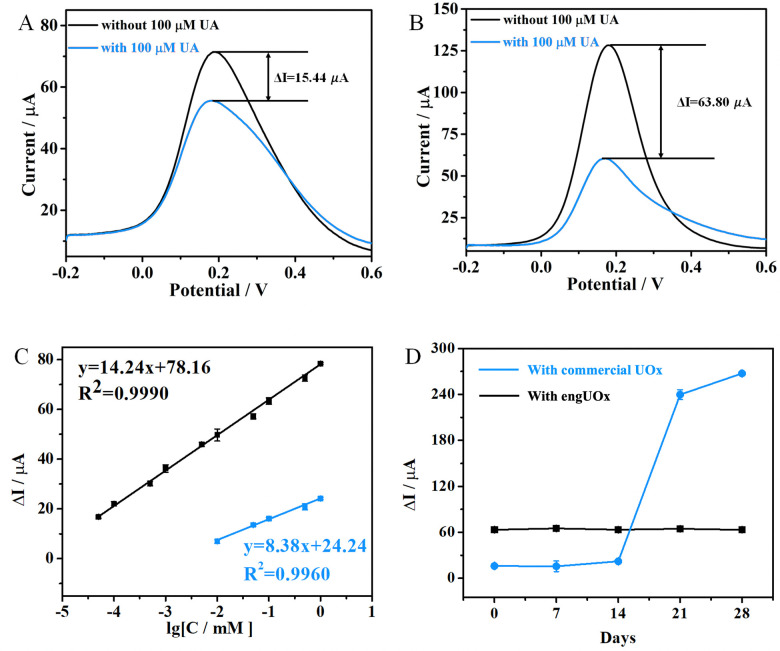
Superior biosensing performance of engUOx compared with commercial UOx. SWV current signal difference of the biosensor constructed with commercial UOx (A) and engUOx (B). (C) The calibration plot between the Δ*I* (−0.251 V *vs.* Ag/AgCl) and the logarithm values of UA concentrations obtained for engUOx (black) and commercially available UOx (blue). (D) Comparison of the life-time stability of our biosensing platform using either engUOx or commercial UOx; stability was monitored as SWV current responses. The error bars denote standard deviations for *n* = 3.

As a result, our biosensor performed better when using engUOx than conventional, commercial UOx (LOD = 5110 nM). Notably, the LOD was thousand-fold lower and the linear range was hundred-fold higher for the biosensor with engUOx compared with the same with commercial UOx. The sensitivity of UA biosensor with engUOx (14.24 μA[logC_UA_]^−1^) was almost two-fold higher than the one with commercial UOx (8.38 μA[logC_UA_]^−1^), which allows for a more precise quantification of UA with the former (Fig. 4C). Finally, we also compared the life-time of our biosensing platform using either engUOx or commercial UOx. The biosensor fabricated with engUOx exhibited at least twice longer life-time than that of the biosensor fabricated with commercial UOx (Fig. 4D). Therefore, our biosensor for UA detection based on engUOx has favorable storage properties that makes it suitable for real-world application.

In summary, we have designed and developed a biosensing platform for the highly sensitive and reliable detection of UA within a wide linear range of concentrations. This work shows how the performance of electrochemical biosensors can be improved by implementing engineered enzymes. It also reiterates the effectiveness of biochemical technique in advancing the biosensing field and provides a robust strategy for the evolution of similar sensors.

Z. W. and Z. Z. thank the Chinese Scholarship Council (CSC) for PhD scholarship funding. F. G. M. thanks the NWO Sector Plan for Physics and Chemistry for funding.

## Conflicts of interest

There are no conflicts to declare.

## Supplementary Material

CC-059-D3CC01869E-s001
