# Supplementary material for: A high-performance electrochemical biosensor using an engineered urate oxidase
Source: Chem Commun (Camb). 2023 May 30;59(52):8071–4. doi: 10.1039/d3cc01869e (PMC10297830; doi:10.1039/d3cc01869e)
Supplement: CC-059-D3CC01869E-s001 [file CC-059-D3CC01869E-s001.pdf]

Electronic Supplementary Material (ESI) for ChemComm.
This journal is © The Royal Society of Chemistry 2023

Supplementary material

A high-performance electrochemical biosensor using an engineered urate oxidase

Zheng Wei, Tanja Knaus, Yuxin Liu, Ziran Zhai, Andrea F. G. Gargano, Gadi Rothenberg,

Ning Yan, and Francesco G. Mutti*

van' t Hoff Institute for Molecular Sciences, University of Amsterdam, Science Park 904, 1098 XH, Amsterdam, The Netherlands

* Corresponding author

E-mail: f.mutti@uva.nl

Table of Contents

1. Experimental section 3

1.1. Materials and equipment 3

1.2. Expression and purification of the enzyme variant UOx R298C 3

1.3. Activity test for UOx R298C 4

1.4. Electrodeposition of gold nanoparticle (AuNPs) on glass-carbon electrode (GCE) 4

1.5. Fabrication of the UA biosensing platform under the optimized condition 5

1.6. General procedure for electrochemical measurements using UA biosensor 5

1.7. Selectivity property of UA biosensor 5

1.8. Stability test of UA biosensor 6

1.9. Micro-flow SEC-MS to study GSH binding to BSA. 6

1.10. Sequence alignment between engUOx and commercially available UOx from Candida sp. 7

Figure S1. 8

Scheme S1 9

Figure S2 10

Figure S3 11

Figure S4 12

Figure S5 13

Figure S6 14

Table S1. Comparison of the detection performance of reported UA biosensor. 15

Table S2. The current response of interfering chemicals 16

Reference 17

1. Experimental section

1.1. Materials and equipment

Hydrogen tetrachloroaurate (III) hydrate ($HAuCl_4 \cdot 3H_2O$), ascorbic acid (AA, 99.7%), $K_2Fe(CN)_6$, uric acid (UA), Ammonium acetate, Glutathione (GSH), urea, N-hydroxysuccinimide (NHS), dopamine (Dop), cysteine (L-Cys) and bovine serum albumin (BSA) and commercial urate oxidase from *Candida* sp. were purchased from Sigma-Aldrich (Darmstadt, Germany). KCl and KH_2PO_4 were purchased from Merck (Darmstadt, Germany). D-Glucose was purchased from Fisher. Thioglycolic acid and N-(3-Dimethylaminopropyl)-N′-ethylcarbodiimide hydrochloride (EDC) were purchased from TCI Europe. K_2HPO_4 and NaCl were purchase from Carl Roth. All solutions were prepared using deionized water. The phosphate buffer saline (PBS) contains 0.1 M NaCl and 10 mM phosphate buffer (pH = 7.4).

Square-wave voltammetry (SWV) curves, cyclic voltammetry, and electrochemical impedance spectroscopy (EIS) were measured with the Reference 600 electrochemical station (Gamry Instruments Inc, USA). A three-electrode system was made up of a glassy carbon electrode (GCE, 4 mm in diameter) as the working electrode, an Ag/AgCl electrode (saturated KCl) as reference electrode, and a Pt wire as the counter electrode. Fourier-transform infrared (FT-IR) spectrum was obtained by the Nicolet™ iS50 FTIR Spectrometer (Thermo Fisher Scientific inc., USA). Scanning electron microscope (SEM) images were determined by JSM-7001F scanning electron microscope. Energy-dispersive X-ray spectroscopy (EDS) pictures were obtained by JEOL ISM-IT100. The micro flow SEC-MS setup was performed on the UltiMate RSLCnano system (Thermo Fisher Scientific, Breda, The Netherlands) and QExactive-Plus Hybrid Quadrupole-Orbitrap mass spectrometer (Thermo Fisher Scientific, Bremen, Germany).

1.2. Expression and purification of the enzyme variant UOx R298C

The synthetic gene encoding for the urate oxidase variant R298C from *Bacillus sp.* TB-90[1-3] (in the main manuscript referred as engUOx) was subcloned into a pET21a(+) plasmid between NdeI and XhoI restriction sites. The sequence contained a flexible linker (LQNAPAHG) before the XhoI site. *E. coli* BL21(DE3) cells were used as the host organism.

The UOx R298C expression and purification were performed according to the following procedure: For recombinant expression, 800 mL of lysogeny broth (LB) medium supplemented with ampicillin (100 µg/mL) were inoculated with 15 mL of an overnight culture harboring the desired plasmid DNA. Cells were grown at 37 °C until an OD_{600} of 0.8-0.9 was reached, and the protein expression was induced by the addition of IPTG (0.5 mM). Protein expression was carried out overnight at 25 °C. After harvesting of the cells (4 °C, 8×10^3 rpm, 10 min), the remaining cell pellet was resuspended in lysis buffer (50 mM KH_2PO_4, 300 mM NaCl, 10 mM imidazole, pH 8.0) prior to cell disruption by ultrasonication. The protein purification was performed by Ni-NTA affinity chromatography using pre-packed Ni-NTA HisTrap HP columns (GE Healthcare), previously equilibrated with lysis buffer. After loading of the filtered lysate, the column was washed with sufficient amounts of washing buffer (50 mM KH_2PO_4, 300 mM NaCl, 25 mM imidazole, pH 8.0), and bound protein was recovered with elution buffer (50 mM KH_2PO_4, 300 mM NaCl, 200 mM imidazole, pH 8.0). After SDS-PAGE, fractions containing the desired proteins in a sufficient purity (>90%) were pooled and dialyzed against 50 mM K_2HPO_4/KH_2PO_4 buffer (pH 8) overnight and concentrated using Centripreps (Millipore). The purified enzyme solution was stored at -80 °C as aliquots after shock-freezing in liquid nitrogen. The final concentration of the protein was determined at 280 nm (ε_{280} = 36390 M^{-1} cm^{-1}). A typical protein yield of 75 mg/L cell culture was obtained.

1.3. Activity test for UOx R298C

The enzyme was tested for its activity by following the decrease in the absorbance of uric acid at 291 nm (ε = 12200 M^{-1} cm^{-1}) as described in reference[2] at room temperature. The reaction mixture contained buffer (KPi, 50 mM, pH 8), uric acid (120 µM, added as a 5 mM stock in buffer) and enzyme (0.92 and 0.46 µM, respectively). The enzyme had a k_{cat} value of 23 min^{-1} under these conditions.

1.4. Electrodeposition of gold nanoparticle (AuNPs) on glass-carbon electrode (GCE)

Before modification, the GCE was polished with polishing film and washed by sonication in ultrapure water and ethanol for 1 min, respectively. The electrodepositing solution was prepared by using a 0.1 M KCl solution to dilute a 100 mM $HAuCl_4 \cdot 3H_2O$ solution to obtain a 1 mM $HAuCl_4 \cdot 3H_2O$ solution[4]. Subsequently, the electrodeposition of AuNPs on GCE was performed by cyclic voltammetry

(CV) technique in the range between 0.2 and 1 V with a scan rate of 50 mV s^{-1} in the above electrodepositing solution. Finally, the AuNPs deposited GCE (AuNPs/GCE) was washed by ultrapure water to remove the residual solution and then dried in air at room temperature.

1.5. Fabrication of the UA biosensing platform under the optimized condition

20 μL of a 2 mM aqueous solution of thioglycolic acid were incubated on AuNPs/GCE at 25 °C for 1 h and then washed with deionized water. Next, 50 μL of a 400 mM EDC solution was mixed with a 100 mM NHS solution, and the resulting mixture was dropped on the thioglycolic acid/AuNPs/GCE at 25 °C for 30 min to activate the carboxylic group of thioglycolic acid. The modified electrode was washed several times by ultrapure water to remove the residual solution. Then, 20 μL of a 1 mg ml^{-1} UOx R298C solution was dropped on the surface of the modified electrode at 37 °C. After 2 h, the modified electrode was washed with deionized water to remove the excess of UOx R298C. Finally, 20 μL of a 200 μM GSH solution was dropped on the biosensing interface; then, the electrode was kept at 37 °C for 60 min. After that, the electrode was washed with deionized water.

1.6. General procedure for electrochemical measurements using UA biosensor

10 μL of a BSA solution was mixed with 10 μL of a UA solution at different concentrations. The mixture was incubated on the biosensing interface at 37 °C for 2 h. After being rinsed by deionized water, the resulted electrodes were measured in a 5 mM $[Fe(CN)_6]^{3-/4-}$ and 0.1 M KCl aqueous solution by square wave voltammetry (SWV) in the range from -0.2 to 0.6 V with pulse amplitude of 25 mV and an increased potential of 4 mV.

1.7. Selectivity property of UA biosensor

10 μL of interferent solution and UA solution were dropped on the constructed UA biosensing platform, respectively. Then, 0.1% BSA solution was incubated with the above solution on the electrodes for 2 h at 37 °C. The concentrations of interferents were: AA (1 mM), dopamine (1 mM), Glu (1 mM), GSH (1 mM), L-Cys (1 mM), urea (1 mM) and thioglycolic acid (1 mM), respectively. The mixture contains all these interferents (1 mM, each) and UA (0.1 mM). Finally, the electrochemical

signal was read out in a 5 mM $[Fe(CN)_6]^{3-/4-}$ and 0.1 M KCl solution by square wave voltammetry (SWV) in the range from -0.2 to 0.6 V with pulse amplitude of 25 mV and an increased potential of 4 mV. After each incubation steps, the electrodes were washed with ultra-pure water.

1.8. Stability test of UA biosensor

The constructed UA biosensing platform was stored successfully at room temperature (22 °C) for 28 days. After every 7 days, 20 μL of the UA and BSA mixed solution were incubated on the biosensing interface and the catalytic reactions were processed at 37 °C for 2 h. Finally, the electrochemical signal was read out in a 5 mM $[Fe(CN)_6]^{3-/4-}$ and 0.1 M KCl aqueous solution by square wave voltammetry (SWV) in the range from -0.2 to 0.6 V with pulse amplitude of 25 mV and an increased potential of 4 mV.

1.9. Micro-flow SEC-MS to study GSH binding to BSA.

The binding of GSH to BSA under oxidative conditions was studied using native microflow SEC-MS methods.[12] The samples were prepared by incubation of aqueous solutions of H_2O_2 (250 μL, 1 mM), BSA (250 μL, 10 mg/mL) and GSH (500 μL, 200 μM) in Eppendorf tubes, at 37 °C, for 2 h and in an orbital shaker (170 rpm). Therefore, the final volume was 1 mL (final concentrations of BSA, H_2O_2 and GSH in the reaction solution were 2.5 mg/ml, 250 μM and 100 μM respectively). A 200 mM ammonium acetate was employed as the dilution solvent when they were analyzed. For the microflow SEC part, the measurement was conducted by TSK gel Super SW3000 (1.0 × 300 mm × 4 μm, TOSOH, Japan) column at a flow rate of 15 μL/min with 200 mM ammonium acetate as mobile phase, and 1 μL as injection volume. The MS spectrum was acquired with HRM-AIF mode. The parameters were as follows. Polarity: positive. In-source CID: 50 eV. Microscans: 10. Resolution: 17500. Maximum injection time: 200 ms. Scan range: 350 to 6000. AGC target: 3×10^6. Capillary temperature: 275 °C. Sheath gas flow: 15. Auxiliary gas flow: 5. Spray voltage: 2.5 kV. S-lens RF level: 200. The MS data was visualized with FreeStyle 1.6 software (Thermo Fisher Scientific). The deconvolution results were carried out with UniDec software (University of Arizona, Phoenix, AZ, USA).[13]

1.10. <u>Sequence alignment between engUOx and commercially available UOx from Candida sp.</u>

Unfortunately, the amino acid sequence of the commercial UOx from *Candida sp.* (Sigma-Aldrich) is not available. Therefore, a structural comparison, even via homology modeling, is not possible. However, judging from past literature, the amino acid sequence of UOx from *Candida sp.* (Sigma-Aldrich) must be similar to the sequence of the UOx from *Candida utilis*.[14] An amino acid sequence alignment between engUOx (i.e., *Bacillus sp.* TB-90)[1,2] and UOx from *Candida utilis*[14] reveals a very low sequence identity.

Sequence 1 is engUOx (i.e. *Bacillus sp.* TB-90).
Sequence 2 is UOx from *Candida utilis*.

The percentage of sequence identity is only 24%.

```
CLUSTAL O(1.2.4) multiple sequence alignment


sequence1      TKHKERVMYYGKGDVFAYRTYLKPLTGVRTIPESPFSGRDHILFGVNVKISVGGTKLLTS     60
sequence2      MSTTLSSSTYGKDNVKFL--------KVKKDPQNP---KKQEVMEATVTCL-LEGGFDTS     48
                . .      ***.:*            *:. *:.*   :.: :: ..*.        : **

sequence1      FTKGDNSLVVATDSMKNFIQKHLASYTGTTIEGFLEYVATSFLKKYSHIEKISLIGE---     117
sequence2      YTEADNSSIVPTDTVKNTILVLAKTTEIWPIERFAAKLATHFVEKYSHVSGVSVKIVQDR     108
               :*:.*** :* **::** *     :     ** *   :** *::****:. :*:

sequence1      -------EIPFETTFAVKNGNRAASELVFKKSRNEYATAYLNMVRNEDNTLNITEQQSGL     170
sequence2      WVKYAVDGKPHDHSFIHEGGEKRITDLYYKRSGD------------------YKLSSAI     149
                        *.: :*  :.*::  ::* :*:* :                   : .*.:

sequence1      AGLQLIKVSGNSFVGFIRDEYTTLPEDSNRPLFVYLNIKWKYKNTEDSFGTNPENY----     226
sequence2      KDLTVLKSTGSMFYGYNKCDFTTLQPTTDRILSTDVDATWVWDNKKIGSVYDIAKAADKG     209
                .* ::* :*. * *: : ::***   ::* * . :: .* :.*.: .   :  :

sequence1      ---VAAEQIRDIATSVFHETETLSIQHLIYLIGRRILERFPQLQEVYFESQ-------NH     276
sequence2      IFDNVYNQAREITLTTFALENSPSVQATMFNMATQILEKACSVYSVSYALPNKHYFLIDL     269
                   . :* *:*: :.*   :: *:*  :: :. :***:  .: .* :          :

sequence1      TWDKIVEEIPESEGKVYTEPCPPYGFQCFTVTQEDLPHENILMFSDEPDHKGALK  331
sequence2      KWKGL----ENDNELFYPSPHPNGLIKCTVVRKEKTKL----------------  303
               .*. :     :.:  .* .* *   ::* .* :*.
```

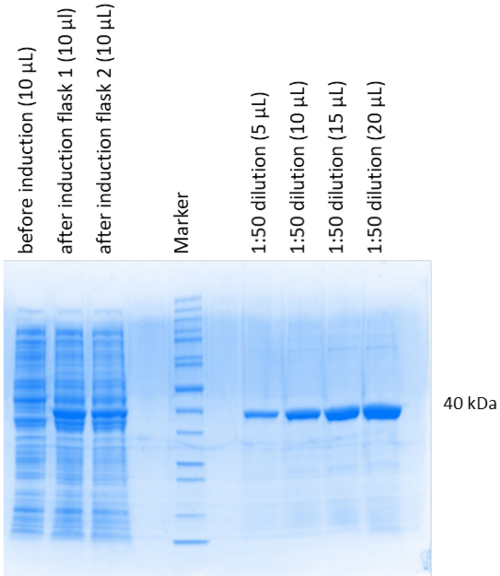

Figure S1. SDS-Page analysis for the expression of UOx R298C (engUOx) and of the purified protein. Marker: PageRuler™ Unstained Protein Ladder (Thermo Fisher Scientific).

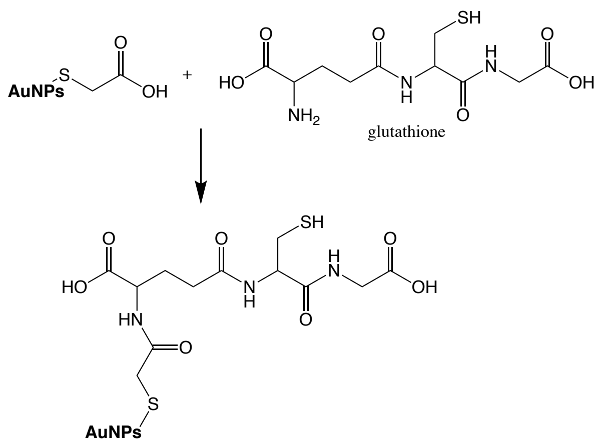

Scheme S1. The mechanism for the formation of the covalent bond between glutathione (-NH_2 group) and thioglycolic acid (-COOH group).

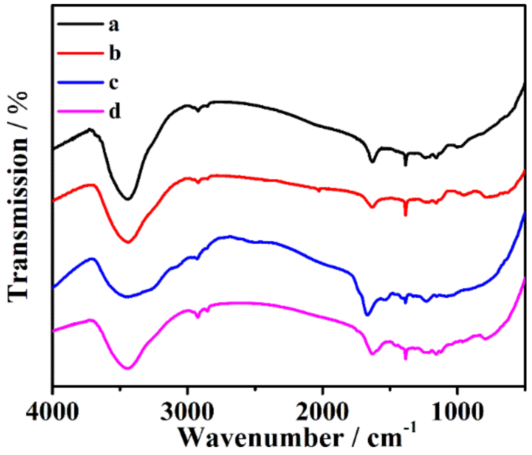

Figure S2. FT-IR spectrum of deposited AuNPs on the electrode (a), immobilized with engUOx (b), incubated with blocking agents (c), and incubated with UA and BSA solution (d).

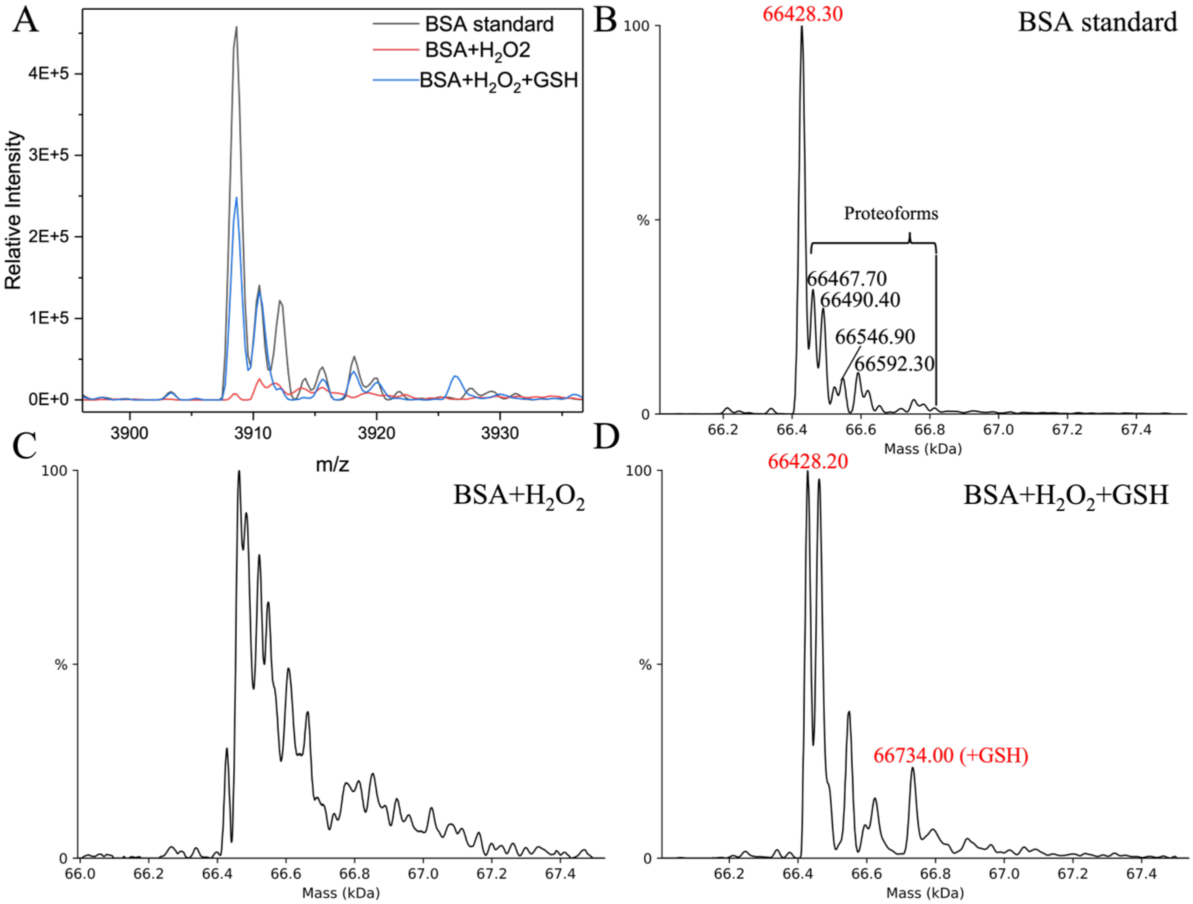

Figure S3. Comparison of the most abundant charge state MS spectrum (+17) for BSA (bovine serum albumin) protein: reference BSA, BSA exposed to H_2O_2, and BSA exposed to both H_2O_2 and GSH (A). Deconvoluted MS spectra of pure BSA (B), BSA reacted with H_2O_2 (C), and BSA reacted with H_2O_2 and GSH (D). In the presence of GSH a peak at 66734.00 Da was observed. With the deconvolution results, the BSA standard has different proteoforms, among which 66428.20 Da was the most abundant. After being treated with H_2O_2, the BSA was oxidized, leading to a complex heterogeneous mixture of oxidized species. In the presence of GSH, a new peak at 66734.00 Da was observed after the reaction, which confirms the linkage between GSH (mass 307.33 Da) and BSA. The error in ppm of the GSH assignment is of 7.10 ppm

$$(\text{error}= \frac{Mass_{theory}-Mass_{observed}}{Mass_{theory}}*10^{\wedge}6= \frac{(66428.20+307.33-2)-66734.00}{66428.20}=7.10 \text{ ppm})$$

which reflects a good accuracy in the modification of a 66 kDa molecule.

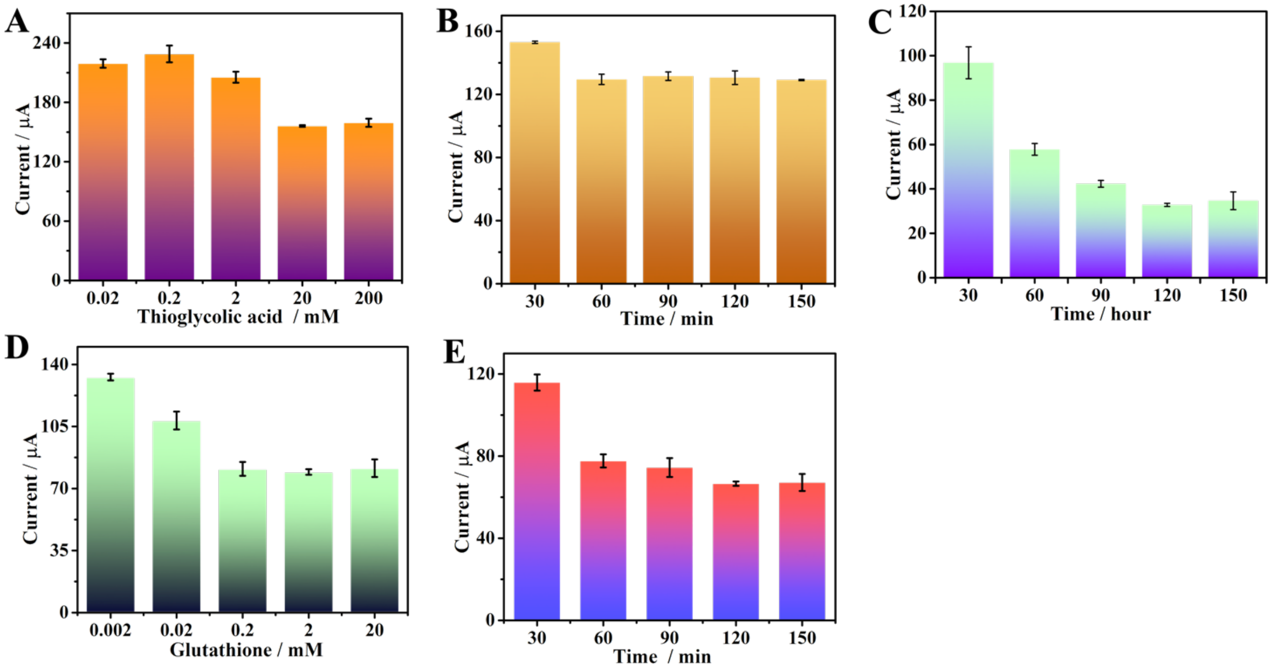

Figure S4. Effect of the concentration of thioglycolic acid (a), incubation time of thioglycolic acid (b), incubation time of engUOx (c), concentration of GSH (d), and catalytic reaction time on the SWV responses. Error bars represent standard deviations of three experiments in parallel.

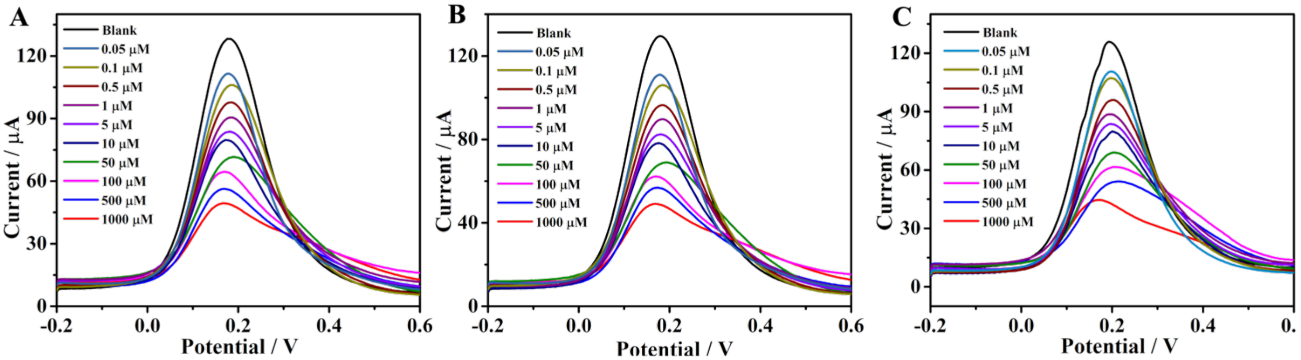

Figure S5. The performance of electrochemical UA biosensor with engUOx. SWV responses of electrochemical detection for UA (three independent determinations).

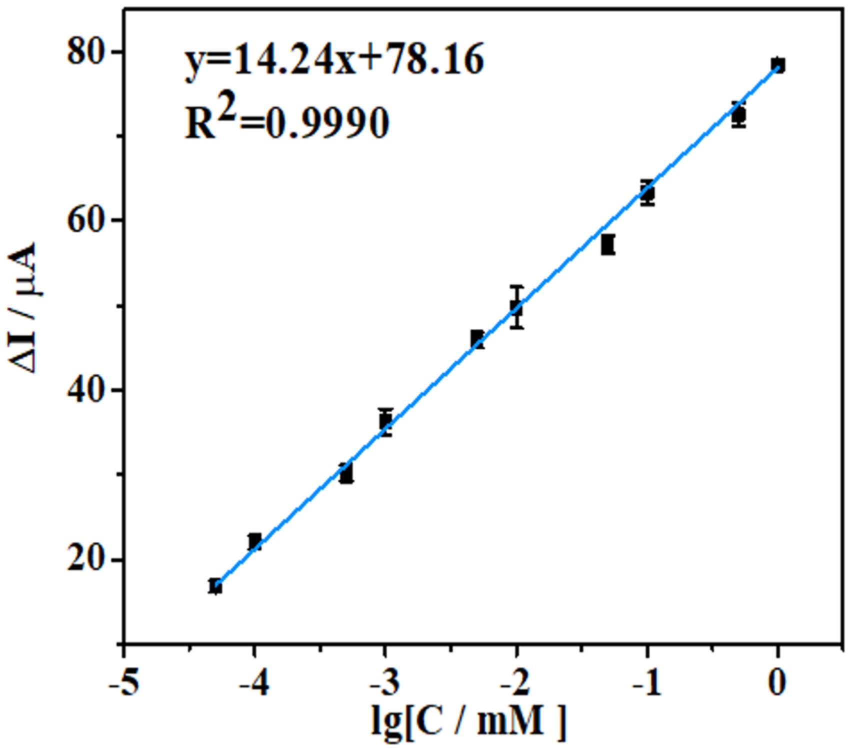

Figure S6. The calibration plot between the ΔI (-0.251 V vs. Ag/AgCl) and the logarithm values of UA concentrations for engUOx (the error bars denote standard deviations for n=3).

Table S1. Comparison of the detection performance of reported UA biosensor.

Entry	Types of sensors	Linear range	LOD	Reference
1	Fluorescent	0.01−400 μM	2.3 nM	Qu et[5].
2	Electrochemical	up to 700 μM	0.066 μM	Jain et[6].
3	Organic electrochemical transistors	50−1000 μM	4.5 μM	Galliani et[7].
4	Electrochemical	25−2500 μM	0.023 μM	Wnag et[8].
5	Electrochemical	50–2000 μM	0.019 μM	Ahmad et[9].
6	Electrochemical	5−100 μM	0.33 μM	Shi et[10].
7	Electrochemical	0.01−0.145 μM	6 nM	Abbas et[11].
8	Electrochemical	0.05−1000 μM	9.16 nM	This work

Table S2. The current response of interfering chemicals

Interference	Con (μM)	Current (μA)	Average (μA)	Standard deviation
Blank	0	129.6<br>128.2<br>125.4	127.7	2.139
Ascorbic acid	1000	113.8<br>112.9<br>113.3	113.3	0.4510
Dopamine	1000	117.3<br>116.5<br>117.6	117.1	0.5690
Glucose	1000	117.9<br>117.7<br>113.8	116.5	2.312
Glutathione	1000	109.8<br>110.9<br>111.4	110.7	0.8190
L-cysteine	1000	115.7<br>116.4<br>116.0	116.0	0.3510
Urea	1000	108.9<br>110.0<br>111.6	110.1	1.358
Thioglycolic acid	1000	107.1<br>108.8<br>112.2	109.3	2.597
Uric acid	100	62.50<br>64.40<br>65.00	63.97	1.305
Mix solution	UA 100<br>Others<br>1000	65.25<br>63.92<br>63.28	64.16	1.010

Reference

1. Hibi, T.; Kume, A.; Kawamura, A.; Itoh, T.; Fukada, H.; Nishiya, Y., Hyperstabilization of Tetrameric *Bacillus* sp. TB-90 Urate Oxidase by Introducing Disulfide Bonds through Structural Plasticity. *Biochemistry* **2016,** *55* (4), 724-32.
2. Hibi, T.; Hayashi, Y.; Fukada, H.; Itoh, T.; Nago, T.; Nishiya, Y., Intersubunit salt bridges with a sulfate anion control subunit dissociation and thermal stabilization of *Bacillus* sp. TB-90 urate oxidase. *Biochemistry* **2014,** *53* (24), 3879-888.
3. Takashio, M.; Chikano, T.; Kamimura, M. Uricase and a method for the preparation thereof. EP0204283B1, 1992.
4. Zhang, D. S.; Li, W. X.; Ma, Z. F., Improved sandwich-format electrochemical immunosensor based on "smart" Sio(2)@polydopamine nanocarrier. *Biosens. Bioelectron.* **2018,** *109*, 171-176.
5. Qu, S.; Li, Z.; Jia, Q., Detection of Purine Metabolite Uric Acid with Picolinic-Acid-Functionalized Metal-Organic Frameworks. *ACS Appl Mater Interfaces* **2019,** *11* (37), 34196-34202.
6. Jain, S.; Verma, S.; Singh, S. P.; Sharma, S. N., An electrochemical biosensor based on novel butylamine capped CZTS nanoparticles immobilized by uricase for uric acid detection. *Biosens Bioelectron* **2019,** *127*, 135-141.
7. Galliani, M.; Diacci, C.; Berto, M.; Sensi, M.; Beni, V.; Berggren, M.; Borsari, M.; Simon, D. T.; Biscarini, F.; Bortolotti, C. A., Flexible Printed Organic Electrochemical Transistors for the Detection of Uric Acid in Artificial Wound Exudate. *Adv. Mater. Interfaces* **2020,** *7* (23), 2001218.
8. Wang, K.; Wu, C.; Wang, F.; Liao, M.; Jiang, G., Bimetallic nanoparticles decorated hollow nanoporous carbon framework as nanozyme biosensor for highly sensitive electrochemical sensing of uric acid. *Biosens. Bioelectron.* **2020,** *150*, 111869.
9. Ahmad, R.; Tripathy, N.; Jang, N. K.; Khang, G.; Hahn, Y. B., Fabrication of highly sensitive uric acid biosensor based on directly grown ZnO nanosheets on electrode surface. *Sens. Actuator B-Chem.* **2015,** *206*, 146-151.
10. Shi, W.; Li, J.; Wu, J.; Wei, Q.; Chen, C.; Bao, N.; Yu, C.; Gu, H., An electrochemical biosensor based on multi-wall carbon nanotube-modified screen-printed electrode immobilized by uricase for the detection of salivary uric acid. *Anal. Bioanal. Chem.* **2020,** *412* (26), 7275-7283.
11. Abbas, M. W.; Soomro, R. A.; Kalwar, N. H.; Zahoor, M.; Avci, A.; Pehlivan, E.; Hallam, K. R.; Willander, M., Carbon quantum dot coated Fe3O4 hybrid composites for sensitive electrochemical detection of uric acid. *Microchem. J.* **2019,** *146*, 517-524.
12. Ventouri, I. K.; Veelders, S.; Passamonti, M.; Endres, P.; Roemling, R.; Schoenmakers, P. J.; Somsen, G. W.; Haselberg, R.; Gargano, A. F. G. Micro-Flow Size-Exclusion Chromatography for Enhanced Native Mass Spectrometry of Proteins and Protein Complexes. *ChemRxiv.* **2022**, doi:10.26434/chemrxiv-2022-pgvsc.
13. Michael T. M.; Andrew J. B.; Erik G. M.; Georg K. A. H.; Justin L. P. B.; Carol V. R.; Bayesian Deconvolution of Mass and Ion Mobility Spectra: From Binary Interactions to Polydisperse Ensembles. *Anal. Chem*. **2015**, 87(8), 4370–4376.
14. Tao, L.; Li, D.; Li, Y.; Shi, X.; Wang, J.; Rao, C.; Zhang, Y., Designing a mutant Candida uricase with improved polymerization state and enzymatic activity. *Protein Eng. Des. Sel.* **2017,** *30* (11), 753-759.
